# Design, Synthesis and Antitubercular Activity of Certain Nicotinic Acid Hydrazides

**DOI:** 10.3390/molecules20058800

**Published:** 2015-05-15

**Authors:** Wagdy M. Eldehna, Mohamed Fares, Marwa M. Abdel-Aziz, Hatem A. Abdel-Aziz

**Affiliations:** 1 Department of Pharmaceutical Chemistry, Faculty of Pharmacy, Egyptian Russian University, Badr City, Cairo 11829, Egypt; E-Mail: ph.fares@yahoo.com; 2 The Regional Center for Mycology and Biotechnology, Al-Azhar University, Cairo 11759, Egypt; E-Mail: marwa2rcmb@yahoo.com; 3 Department of Pharmaceutical Chemistry, College of Pharmacy, King Saud University, P.O. Box 2457, Riyadh 11451, Saudi Arabia; 4 Department of Applied Organic Chemistry, National Research Center, Dokki, Cairo 12622, Egypt

**Keywords:** synthesis, nicotinic acid, hydrazides, antimycobacterial, ADME

## Abstract

Three series of 6-aryl-2-methylnicotinohydrazides **4a**–**i**, *N*′-arylidene-6-(4-bromophenyl)-2-methylnicotino hydrazides **7a**–**f**, and *N*′-(un/substituted 2-oxoindolin-3-ylidene)-6-(4-fluorophenyl)-2-methylnicotinohydrazides **8a**–**c** were synthesized and evaluated for their potential *in vitro* antimycobacterial activity against *M. tuberculosis*. The results showed that isatin hydrazides **8a**–**c** are remarkably more active than the parent hydrazide **4c**. Hydrazides **8b** and **8c** exhibited the highest activity among all the tested compounds (MIC = 12.5 and 6.25 µg/mL, respectively). Compounds **8b** and **8c** were also devoid of apparent cytotoxicity to HT-29, PC-3, A549, HepG2 and MCF-7 cancer cell lines. Besides, **8b** and **8c** showed good drug-likeness scores of 0.62 and 0.41, respectively. Those two isatin hydrazides could offer an excellent framework for future development to obtain more potent antitubercular agents. The SAR study suggested that lipophilicity of the synthesized derivatives is a crucial element that accounts for their antimycobacterial activity. Finally, a theoretical kinetic study was established to predict the ADME of the active derivatives.

## 1. Introduction

The Nineteenth World Health Organization (WHO) Tuberculosis Report indicates that TB is one of the world’s deadliest communicable diseases [1]. It estimates that in 2013 there were 9.0 million new cases and 1.5 million deaths from TB, including 400,000 deaths associated with co-infection with HIV. The highest rates *per capita* occurred in the African Region (25%), while South-East Asia, the Western Pacific and African Regions account for around 81% of all the total cases [1]. The disease is aggravated by the worldwide continuous emergence of multidrug-resistant strains of *M. tuberculosis* (MDR-TB), extensively drug-resistant tuberculosis (XDR-TB) and totally drug-resistant tuberculosis (TDR-TB) [2,3]. The magnitude and extent of drug-resistant strains have increased concern that TB may once again become an incurable disease [4,5]. Moreover, the increasing incidence of the disease in immunocompromised patients along with the longer durations of therapy emphasize the need for new drugs to extend the range of effective TB treatment options [6,7,8].

**Figure 1:**
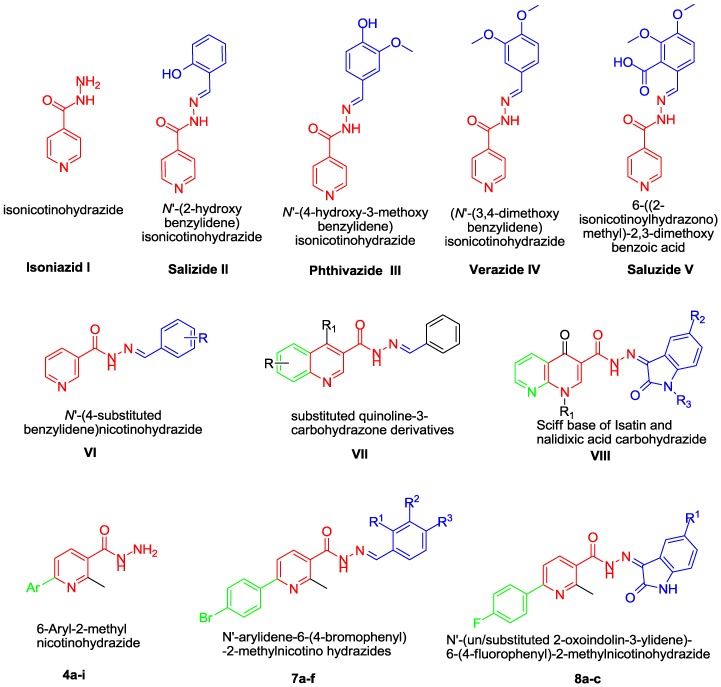
Structures of antitubercular drugs **I**–**VIII** and the target derivatives **4a**–**i**, **7a**–**f** and **8a**–**c**.

TB treatment is tedious, challenging and time-consuming. It requires the administration of appropriate treatment regimens for at least six months via directly observed therapy (DOT) and follow-up support [2]. Treatment regimens require a minimum six months in two separate phases. The duration of phase one is about two months and involves four drugs (isoniazid, rifampicin, pyrazinamide and ethambutol), followed by four months of phase two (using isoniazid plus rifampicin) [9]. However, the present treatment regimen has some limitations such as drug toxicity and intolerance, drug–drug interactions and poor patient adherence due to the lengthy treatment duration [10]. Therefore, there is an urgent need for the development and more efficient evaluation of new TB drugs and shorter treatment regimens. Isoniazid (INH, Figure 1), a critical frontline drug in TB treatment discovered by Dogmagk, is a prodrug that requires activation *in vivo* by mycobacterial catalase peroxidase (KatG) [11,12]. INH exerts its anti-tubercular activity *via* interference with the synthesis of mycolic acid, one of the essential chemical pathways responsible for the formation of cell walls in *M. tuberculosis* [13].

The enzymatic acetylation of isoniazid by *N*-acetyltransferase (NAT) represents a major metabolic pathway for isoniazid in humans [14], so blocking acetylation *via* chemical modification of the hydrazine unit with a functional group, while preserving potent antimycobacterial action, has the potential to counterbalance the known side effects of INH, improve clinical outcomes and reduce the emergence of acquired isoniazid resistance in patients. Subsequently, numerous studies have pointed out the importance of developing novel INH hydrazides as promising anti-tubercular agents (Figure 1) [15,16,17,18,19,20,21,22].

Recently, Narang *et al.* [23] developed a novel series of nicotinic acid hydrazide derivatives as potential antimycobacterial agents with a general structure represented by **VI** (Figure 1). The results showed that the presence of lipophilic electron-withdrawing halogen groups at the *para* position of the phenyl ring improved the antimycobacterial activity. Concerning the related fused pyridine heterocycles, Adhikari and co-workers reported two studies on the design, synthesis and biological evaluation of two different series of new quinoline-3-carbohydrazone derivatives **VII**, (Figure 1), as potential antimycobacterial agents [24,25]. Aboul-Fadl *et al.* [26] also explored the anti-tubercular activity of Schiff bases **VIII** of nalidixic acid-3-carbohydrazides and isatins. On the other hand, isatin-based compounds are known to exhibit excellent anti-TB properties [27,28,29,30].

In this work, the aforementioned findings motivated us to synthesize three series of nicotinic acid hydrazide derivatives **4a**–**i**, **7a**–**f** and **8a**–**c** with the aim of obtaining new antimycobacterial agents. The first series comprises different nine 6-aryl-2-methylnicotinohydrazides with free hydrazine units in a similar fashion to INH. Subsequently, two derivatives (compounds **4c** and **4e**) with considerable lipophilicity (LogP = 1.53 and 2.17, respectively) were chosen for further chemical modification. The non-classical ring opening bioisosterism for structures **VI** and **VIII** was adopted to develop a series of aldehyde hydrazides **7a**–**f** and a series of isatin hydrazides **8a**–**c**.

The *in vitro* cytotoxic activity of subset of compounds was evaluated against HT-29, PC-3, A549, HepG2 and MCF-7 cancer cell lines to determine the toxicity of these agents. In addition, we describe an ADME study and SAR description in order to explore the structural requirements controlling these observed antitubercular activities.

## 2. Results and Discussion

### 2.1. Chemistry

**Scheme 1:**
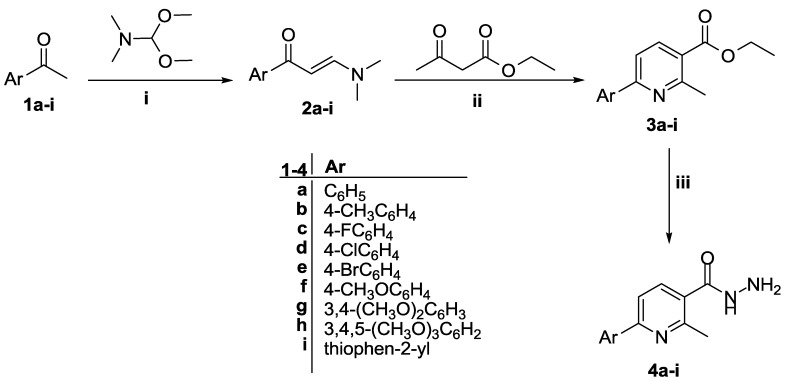
Synthesis of nicotinic acid hydrazides **4a**–**i**.

**Scheme 2:**
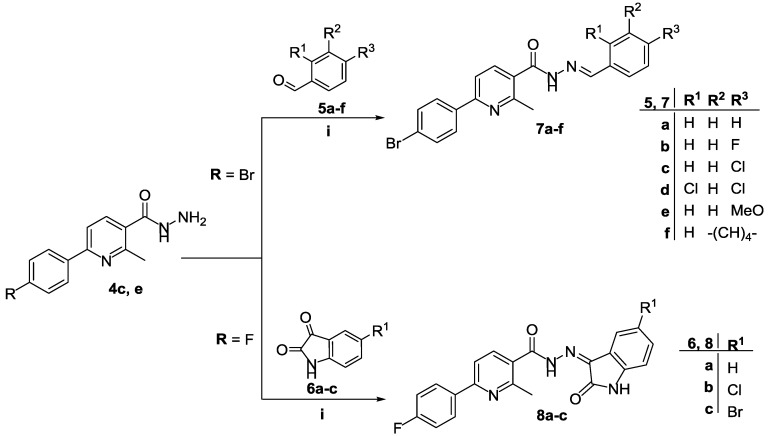
Synthesis of nicotinic acid hydrazides **7a**–**f** and **8a**–**c**.

The synthetic pathways employed to prepare the new targeted derivatives are depicted in Scheme 1 and Scheme 2. In a one-pot three-component heterocyclocondensation process, ethyl 2-methyl-6-arylnicotinates **3a**–**i** was obtained via the reaction of enaminones **2a**–**i** with ethyl acetoacetate and ammonium acetate in refluxing acetic acid. Preparation of the nicotinic acid hydrazides **4a**–**i** in 79%–90% yield was achieved via the hydrazinolysis of ester derivatives **3a**–**i** with refluxing hydrazine hydrate (Scheme 1).

*Reagents and conditions*: (i) xylene, reflux 8h; (ii) NH_4_OAc/AcOH/reflux 5 h; (iii) NH_2_NH_2_·H_2_O/reflux 3 h.

The IR spectra of hydrazides **4a**–**i** showed absorption bands due to the carbonyl group in the 1635–1664 cm^−1^ region, in addition to peaks in the region from 3187 to 3414 cm^−1^, assigned to the NH and NH_2_ groups. The ^1^H-NMR spectra of **4a**–**i** showed two singlet D_2_O-exchangeable signals attributable to NH and NH_2_ protons in the δ 9.58–9.62 and 4.52–4.60 ppm region, respectively, while the methyl (-CH_3_) protons appeared as singlets around δ 2.59–2.61 ppm. Furthermore, the ^13^C-NMR spectra of **4f**–**h** showed signals resonating around δ 167 ppm attributable to the carbons of carbonyl groups, while the carbons of the methyl groups appeared in the δ 23.53–23.59 ppm range.

While the hydrazide **4e** was reacted with different aldehydes **5a**–**f**, the hydrazide **4c** was reacted with three isatins **6a**–**c** in ethanol in the presence of a catalytic amount of glacial acetic acid to furnish the target derivatives **7a**–**f** and **8a**–**c**, respectively (Scheme 2).

The IR spectra of hydrazides **7a**–**f** revealed the presence of two peaks in the 3412–3413 and 1635–1654 cm^−1^ regions, assigned to the NH and carbonyl groups, respectively. The ^1^H-NMR spectra of these compounds revealed D_2_O-exchangeable signals in the δ 11.80–12.05 ppm region which were assigned to NH protons, in addition to the signal of the methine proton (-CH=N-) in the δ 8.06–8.96 ppm region. Furthermore, the ^13^C-NMR spectra of **7a**–**f** showed two signals resonating at δ 23.41–23.47 and δ 162.00–170.39 ppm attributable to the methyl (CH_3_) and carbonyl (=C-C=O) carbons, respectively.

*Reagents and conditions*: i, EtOH/AcOH (catalytic)/reflux 4 h.

### 2.2. Biological Evaluation

#### 2.2.1. *In Vitro* Antimycobacterial Activity

Biological assays were performed at the Regional Center for Mycology and Biotechnology (RCMB), Al-Azhar University (Cairo, Egypt). Target compounds **4a**–**i**, **7a**–**f** and **8a**–**c** were evaluated *in vitro* for their anti-tubercular activity against *M. tuberculosis* (RCMB 010126) using the microplate Alamar blue assay (MABA) [31]. Isoniazide and pyrazinamide were used as reference drugs. The results as percent inhibition and minimum inhibitory concentration (MIC) are presented in Table 1.

With regard to the issue of activity, valuable information about cell wall structure, host-pathogen interactions and drug targets provide opportunities for rational drug design strategies focused on drug lipophilicity. Increases in lipophilic character, however, result in changes in pathways of diffusion across the cell wall, enhancing the contribution of diffusion through the lipid domain, so increasing the lipophilicity of an antimycobacterial agent enhances its efficacy [32,33,34,35]. The calculated lipophilicity (miLogP) values are listed in Table 1.

Drug-likeness model scores were computed for all the compounds using the MolSoft software and are presented in Table 1. Drug-likeness models help to optimize the pharmacokinetic and pharmaceutical properties, for example, solubility, chemical stability, bioavailability and distribution profile of compounds [36]. Counterparts having zero or negative values should not be considered as drug-like candidates. Compounds **7f** and **8a**–**c** possessed the maximum drug-likeness score ranging from 0.41 to 0.88.

**Table 1:** Antitubercular activities, LogP measurements and drug-likeness model scores of nicotinic acid hydrazide derivatives. 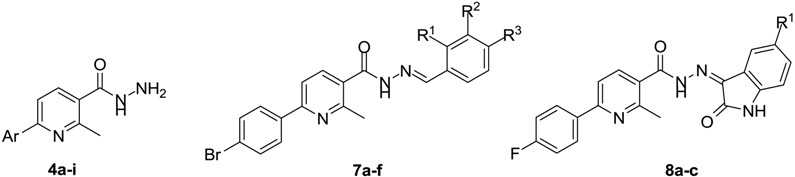

Compound	Ar	R^1^	R^2^	R^3^	Mean of Inhibition %	MIC (µg/mL)	LogP ^a^	Drug-Likeness Model Score ^b^	
**4a**	C_6_H_5_				42.52 ± 0.63	25	1.36	−0.1	
**4b**	4-CH_3_C_6_H_4_				36.33 ± 0.58	25	1.81	−0.36	
**4c**	4-FC_6_H_4_				NA	NA	1.53	−0.06	
**4d**	4-ClC_6_H_4_				12.45 ± 0.58	100	2.04	0.05	
**4e**	4-BrC_6_H_4_				20.63 ± 0.63	50	2.17	−0.25	
**4f**	4-CH_3_OC_6_H_4_				42.63 ± 0.16	25	1.42	−0.25	
**4g**	3,4(CH_3_O)_2_C_6_H_3_				22.63 ± 0.16	50	1.01	0.13	
**4h**	3,4,5(CH_3_O)_3_C_6_H_2_				18.32 ± 0.72	50	0.99	0.33	
**4i**	thiophen-2-yl				22.63 ± 0.20	50	1.15	−0.10	
**7a**		H	H	H	NA	NA	4.95	0.12	
**7b**		H	H	F	NA	NA	5.11	0.00	
**7c**		H	H	Cl	NA	NA	5.63	0.09	
**7d**		Cl	H	Cl	13.57 ± 0.72	100	6.23	0.13	
**7e**		H	H	MeO	NA	NA	5.01	0.09	
**7f**		H	-(CH)_4_-		14.32 ± 0.58	100	6.13	0.46
**8a**		H			31.44 ± 0.58	25	3.54	0.88	
**8b**		Cl			52.63 ± 0.58	12.5	4.19	0.62	
**8c**		Br			77.42 ± 0.93	6.25	4.32	0.41	
**Pyrazinamid**					93.25 ± 0.63	3.21			
**Isoniazide**					-	0.75			

^a^: Calculated by [37]; ^b^: Calculated by [38]; NA: No Activity (>100 µg/mL).

As shown in Table 1, compound **8c** emerged as the most potent analog with good antimycobacterial activity (MIC = 6.25 µg/mL). Compound **8b** also possessed reasonable activity with a MIC value of 12.5 µg/mL. Besides, derivatives **4a**, **4b**, **4f** and **8a** displayed moderate activity (MIC = 25 µg/mL). On the other hand, compounds **4d**, **4e**, **4g**–**i**, **7d** and **7f** exhibited modest antimycobacterial activity with MIC values ranging from 50 to 100 µg/mL.

#### 2.2.2. Structure Activity Relationships (SAR)

Observing the results, we could deduce valuable data about the structure activity correlations of the tested compounds. Firstly, we explored the impact of substitution of the 4-position of the phenyl group in the first series compounds **4a**–**i**. Incorporation of an unsubstituted phenyl group led to compound **4a** with good activity against *M. tuberculosis* (MIC = 25 µg/mL). Introduction of a fluorine atom in the 4-position (compound **4c**) led to complete loss of activity, suggesting that the presence of a strongly electron-withdrawing group is not favorable to the activity. Meanwhile, compounds **4d** and **4e** bearing more lipophilic chlorine and bromine substituents at the same position, elicited better activity (MIC = 50 and 100 µg/mL, respectively) than that of analog **4c**. Thence, the order of activities of the halogenated members in the first series, decreased in the order of Br > Cl > F, indicating that the increased lipophilicity is a crucial element for the antitubercular activity. Conversely, substitution at the 4-position with electron-donating groups as methyl and methoxy groups (compounds **4b** and **4f**) retained the activity at MIC = 25 µg/mL. Interestingly, di- and trimethoxy substitutions (compounds **4g** and **4h**) led to decrease in the lipophilicity with subsequent decrease in the antimycobacterial activity (MIC = 50 µg/mL). Furthermore, the bioisosteric replacement of the phenyl group with a 2-thienyl group (compound **4i**) led to a decrease in the activity.

Considering the aldehyde hydrazides of the second series **7a**–**f**, antimycobacterial activity was only observed in counterparts **7d** and **7f** (MIC = 100 µg/mL). The remaining hydrazides exhibited no activity. Noteworthily, compounds **7d** and **7f** are the most lipophilic analogs in this series with LogP values of 6.23 and 6.13, respectively.

Finally, the effect of substitution of the 5-position of the incorporated isatin moiety in hydrazides **8a**–**c** was investigated. Compound **8a** with an unsubstituted isatin moiety showed good anti-TB activity (MIC = 25 µg/mL). The introduction of a chlorine substituent at the 5-position (compound **8b**) resulted in increased activity with MIC = 12.50 µg/mL. Moreover, incorporation of a bromine atom (compound **8c**) caused a remarkable increase of activity against *M. tuberculosis* (MIC = 6.25 µg/mL). To summarize, the order of activities of the isatin hydrazides of the third series decreased in the order Br > Cl > H, confirming the importance of lipophilicity for the antimycobacterial activity.

In general, the active members in the second series, compounds **7d** and **7f**, are less active than their parent compound **4e**, indicating that condensation of free hydrazides with aldehydes results in a sharp decrease in activity, whilst, the compounds of the third series are remarkably more potent than their parent compound **4c**, hinting that condensation with isatins greatly enhances the antitubercular activity.

#### 2.2.3. *In Vitro* Cytotoxicity

*In vitro* cytotoxicity of the most active antitubercular compounds **8a**–**c** was examined against HT-29 colon cancer and PC-3 prostate cancer cell lines using a sulforhodamine B (SRB) colorimetric assay as described by Skehan *et al*. [39]. Besides, these compounds were evaluated in a previous study for their cytotoxicity against HepG-2 liver cancer, A-549 human lung cancer and MCF-7 breast cancer cell lines [40]. Doxorubicin was included in the experiment as a reference cytotoxic compound. The results were expressed as growth inhibitory concentration (IC_50_) values which represent the compound concentration required to produce a 50% inhibition of cell growth after 72 h of incubation compared to untreated control (Table 2). Interestingly, none of the tested compounds displayed any significant cytotoxicity, thereby providing a high therapeutic index.

**Table 2:** Levels of cytotoxicity induced by hydrazides **8a**–**c** on different cell lines.

Compound	IC_50_ (µM)				
	HT-29	PC-3	A549	HepG2	MCF-7
**8a**	>200	>200	>200	>200	>200
**8b**	>200	>200	>200	>200	>200
**8c**	>200	>200	>200	>200	>200
**Doxorubicin**	7.3 ± 1.11	6.5 ± 1.07	7.6 ± 1.37	6.9 ± 2.05	6.1 ± 1.95

### 2.3. ADME Study

The ADME of the biologically active counterparts **4a**, **4b**, **4d**–**i**, **7d**, **7f** and **8a**–**c** was predicted via a theoretical kinetic study performed by means of the Discovery Studio software (Table 3). Both AlogP98 and PSA_2D descriptors were calculated to evaluate the lipophilicity and polar surface area. Also, solubility, absorption and CYP2D inhibition levels were predicted. Active members of the first series were expected to have good solubility, while compounds **7d** and **7f** showed very low solubility levels and compounds **8a**–**c** showed low solubility levels. All the examined compounds showed good absorption levels, except compound **7d** that displayed a moderate absorption level. Finally, with exception to compounds **4b**–**f**, all members were predicted to be CYP2D non-inhibitors.

**Table 3:** Computer aided ADME study of the active derivatives.

Compound	Alog*P*98 ^a^	PSA_2D ^b^	Solubility ^c^	Solubility Level ^d^	Absorption Level ^e^	CYP2D6 ^f^	CYP2D6 Probability ^g^
**4a**	1.419	67.912	−2.578	3	0	0	0.306
**4b**	1.905	67.912	−3.087	3	0	1	0.772
**4d**	2.083	67.912	−3.496	3	0	1	0.831
**4e**	2.167	67.912	−3.57	3	0	1	0.712
**4f**	1.402	76.842	−2.838	3	0	1	0.722
**4g**	1.386	85.772	−3.101	3	0	0	0.366
**4h**	1.37	94.702	−3.353	3	0	0	0.316
**4i**	1.145	67.912	−2.546	3	0	0	0.158
**7d**	5.839	52.695	−6.834	1	1	0	0.287
**7f**	5.419	52.695	−6.647	1	0	0	0.306
**8a**	3.048	82.806	−4.835	2	0	0	0.485
**8b**	3.713	82.806	−5.608	2	0	0	0.415
**8c**	3.797	82.806	−5.682	2	0	0	0.495

^a^: Lipophilicity descriptor; ^b^: Polar surface area; ^c^: Solubility parameter.(0~−2 = optimal, −2~−4 = good, −4~−6 = low, −6~−8 = very low); ^d^: Solubility level. (0 = extremely low, 1 = very low but possible, 2 = low, 3 = good, 4 = optimal); ^e^: Absorption level. (0 = good, 1 = moderate, 2 = low, 3 = very low); ^f^: CYP2D inhibition. (0 = non inhibitor, 1 = inhibitor); ^g^: CYP2D6 Probability: 0–0.5 = non inhibitor; 0.5–1 = inhibitor.

## 3. Experimental Section

### 3.1. General Information

Melting points were measured with a Stuart melting point apparatus and are uncorrected. Infrared (IR) spectra were recorded as KBr disks using a Perkin Elmer FT-IR Spectrum BX apparatus (Akron, OH, USA). NMR Spectra were recorded on a Bruker AV-500 MHz NMR spectrometer (Billerica, MA, USA). ^1^H-NMR spectra were run at 500 MHz and ^13^C spectra was run at 125 MHz in deuterated dimethylsulfoxide (DMSO-*d_6_*). Chemical shifts are expressed in δ values (ppm) using the solvent peak as internal standard. All coupling constants (*J*) values are given in Hertz. The abbreviations used are as follows: s, singlet; d, doublet; m, multiplet. Microanalyses were carried out using Perkin Elmer PE 2400 CHN Elemental Analyzer and the results were within ±0.4%. Reaction courses and product mixtures were routinely monitored by thin layer chromatography (TLC) on silica gel precoated F_254_ plates Merck (Merck KGaA, Darmstadt, Germany). Unless otherwise noted, all solvents and reagents were commercially available and used without further purification.

### 3.2. Synthesis

#### 3.2.1. Ethyl 2-methyl-6-arylnicotinates **3a**–**i**

To a solution of the appropriate enaminone **2a**–**i** (5 mmol) in glacial acetic acid (15 mL), ethyl acetoacetate (5.5 mmol) and ammonium acetate (40 mmol) were added. The reaction mixture was heated under reflux for 5 h. After cooling and pouring into ice-water, the residue obtained was filtered and washed with petroleum ether then with water and finally crystallized from ethanol [41]. The yields of compounds **3a**–**i** were 80%, 77%, 81%, 84%, 86%, 80%, 785, 76%, 83%, respectively.

#### 3.2.2. 6-Aryl-2-methylnicotinohydrazides **4a**–**i**

A mixture of the appropriate ester **4a**–**i** (5 mmol) and 99% hydrazine hydrate (5 mL) was refluxed for 3 h. The solid product obtained upon cooling was filtered off and recrystallized from dioxan to afford the corresponding 6-aryl-2-methylnicotinohydrazides **4a**–**i**, respectively. The physical properties and spectral data of **4a**–**d** and **4i** were identical with those reported [40,42]. The yields of **4a**–**d** and **4i** were 83, 78, 84, 86 and 84%, respectively.

*6-(4-Bromophenyl)-2-methylnicotinohydrazide* (**4e**). White crystals (yield 90%), m.p. 213–215 °C; IR (KBr, ν cm^−1^): 3194, 3294 (NH, NH_2_) and 1643 (C=O); ^1^H-NMR δ ppm: 2.61 (s, 3H, CH_3_), 4.60 (s, 2H, NH_2_, D_2_O exchangeable), 7.56 (d, *J =* 8.5 Hz, 2H, Ar-H), 7.79 (d, *J =* 8.1 Hz, 1H, H-4 pyridine,), 7.87 (d, *J =* 8.1 Hz, 1H, H-5 pyridine,), 8.14 (d, *J =* 8.5 Hz, 2H, Ar-H), 9.62 (s, 1H, NH, D_2_O exchangeable); Anal. calcd. for C_13_H_12_BrN_3_O (306.16): C, 51.00; H, 3.95; N, 13.72. Found C, 51.13; H, 4.10; N, 13.78.

*6-(4-Methoxyphenyl)-2-methylnicotinohydrazide* (**4f**). White crystals (yield 81%), m.p. 195–197 °C; IR (KBr, ν cm^−1^): 3292, 3350 (NH, NH_2_) and 1635 (C=O); ^1^H-NMR δ ppm: 2.59 (s, 3H, CH_3_), 3.83 (s, 3H, OCH_3_), 4.52 (s, 2H, NH_2_, D_2_O exchangeable), 7.05 (d, *J =* 8.5 Hz, 2H, Ar-H), 7.70 (d, *J =* 8.1 Hz, 1H, H-4 pyridine), 7.73 (d, *J =* 8.1 Hz, 1H, H-5 pyridine), 8.07 (d, *J =* 8.5 Hz, 2H, Ar-H), 9.58 (s, 1H, NH, D_2_O exchangeable); ^13^C-NMR δ ppm: 23.53, 55.72, 114.61, 116.55, 128.54, 129.00, 130.96, 136.78, 155.89, 156.03, 160.85, 167.78; Anal. calcd. for C_14_H_15_N_3_O_2_ (257.12): C, 65.35; H, 5.88; N, 16.33. Found C, 65.54; H, 5.93; N, 16.53.

*6-(3,4-Dimethoxyphenyl)-2-methylnicotinohydrazide* (**4g**). White crystals (yield 85%), m.p. 204–205 °C; IR (KBr, ν cm^−1^): 3187, 3414 (NH, NH_2_) and 1648 (C=O); ^1^H-NMR δ ppm: 2.60 (s, 3H, CH_3_), 3.83 (s, 3H, OCH_3_), 3.86 (s, 3H, OCH_3_), 4.53 (s, 2H, NH_2_, D_2_O exchangeable), 7.06 (d, *J =* 8.5 Hz, 1H, Ar-H), 7.68–7.74 (m, 3H, Ar-H), 7.81 (d, *J =* 8.5 Hz, 1H, Ar-H), 9.59 (s, 1H, NH, D_2_O exchangeable); ^13^C-NMR δ ppm: 23.59, 56.03, 110.41, 112.19, 116.81, 119.95, 128.76, 131.15, 136.71, 149.37, 150.57, 155.83, 156.09, 167.77; Anal. calcd. for C_15_H_17_N_3_O_3_ (287.13): C, 62.71; H, 5.96; N, 14.63; O, 16.71, Found C, 62.84; H, 6.11; N, 14.89.

*2-Methyl-6-(3,4,5-trimethoxyphenyl)nicotinohydrazide* (**4h**). White crystals (yield 85%), m.p. 185–187 °C; IR (KBr, ν cm^−1^): 3282, 3310 (NH, NH_2_) and 1664 (C=O); ^1^H-NMR δ ppm: 2.61 (s, 3H, CH_3_), 3.73 (s, 3H, OCH_3_), 3.87 (s, 6H, OCH_3_), 4.55 (s, 2H, NH_2_, D_2_O exchangeable), 7.41 (s, 2H, Ar-H), 7.75 (d, *J =* 8.0 Hz, 1H, H-4 pyridine), 7.89 (d, *J =* 8.0 Hz, 1H, H-5 pyridine), 9.61 (s, 1H, NH, D_2_O exchangeable); ^13^C-NMR δ ppm: 23.58, 56.46, 60.57, 104.53, 117.51, 129.31, 134.02, 136.73, 139.23, 153.61, 155.84, 155.92, 167.66; Anal. calcd. for C_16_H_23_N_3_O_4_ (321.17): C, 59.80; H, 7.21; N, 13.08. Found C, 60.09; H, 7.43; N, 13.22.

#### 3.2.3. General Procedure for Synthesis of *N*′-arylidene-6-(4-bromophenyl)-2-methylnicotinyl Hydrazides **7a**–**f**

To a stirred solution of the hydrazide **4e** (5 mmol) in hot ethanol (20 mL), aldehydes **5a**–**f** (5 mmol) and catalytic amount of glacial acetic acid were added. The reaction mixture was heated under reflux for 4 h. The precipitate formed was collected by filtration while hot, washed with hot ethanol, dried and crystallized from ethanol/DMF to afford compounds **7a**–**f** in 65%–80% yield.

*N′-Benzylidene-6-(4-bromophenyl)-2-methylnicotinohydrazide* (**7a**). White crystals (yield 75%), m.p. 270–272 °C; IR (KBr, ν cm^−1^): 3410 (NH), 1635 (C=O); ^1^H-NMR δ ppm: 2.67 (s, 3H, CH_3_), 7.31–7.49 (m, 4H, Ar-H), 7.72-7.75 (m, 3H, Ar-H), 7.93-8.13 (m, 4H, Ar-H), 8.33 (s, 1H, CH=N), 11.94 (s, D_2_O exch., 1H, –CONH–); ^13^C-NMR δ ppm: 23.47, 117.59, 123.67, 127.11, 127.65, 129.27, 130.73, 132.22, 132.26, 134.58, 137.39, 137.50, 148.39, 155.48, 156.47, 164.38; Anal. calcd. for C_20_H_16_BrN_3_O (393.05): C, 60.93; H, 4.09; N, 10.66. Found C, 61.07; H, 4.30; N, 10.93.

*6-(4-Bromophenyl)-N'-(4-fluorobenzylidene)-2-methylnicotinohydrazide* (**7b**). White crystals (yield 65%), m.p. 252–254 °C; IR (KBr, ν cm^−1^): 3413 (NH), 1647 (C=O); ^1^H-NMR δ ppm: 2.66 (s, 3H, CH_3_), 7.31 (t, *J =* 8.25 Hz, 2H, Ar-H), 7.72 (d, *J =* 8.5 Hz, 2H, Ar-H), 7.80–7.97 (m, 4H, Ar-H), 8.11-813 (m, 2H, Ar-H), 8.33 (s, 1H, CH=N), 11.95 (s, D_2_O exch., 1H, –CONH–); ^13^C-NMR δ ppm: 23.41, 116.34 (^2^*J*_F-C_ = 22.5 Hz), 117.25, 117.58, 123.68, 129.21 (^3^*J*_F-C_ = 7.5 Hz), 129.64, 130.48, 131.21, 132.21, 137.38, 143.79, 147.26, 154.75, 162.70 (^1^*J*_F-C_ = 211.3 Hz), 170.39; Anal. calcd. for C_20_H_15_BrFN_3_O (411.04): C, 58.27; H, 3.67; N, 10.19. Found C, 58.38; H, 3.83; N, 10.25.

*6-(4-Bromophenyl)-N'-(4-chlorobenzylidene)-2-methylnicotinohydrazide* (**7c**). White crystals (yield 80%), m.p. 265–267 °C; IR (KBr, ν cm^−1^): 3413 (NH),1654 (C=O); ^1^H-NMR δ ppm: 2.66 (s, 3H, CH_3_), 7.20 (d, *J =* 8.50 Hz, 1H, Ar-H), 7.54 (d, *J =* 8.50 Hz, 1H, Ar-H), 7.72 (d, *J =* 8.50 Hz, 2H, Ar-H), 7.77 (d, *J* = 8.50 Hz, 2H, Ar-H), 7.98 (d, *J =* 4.5 Hz, 2H, Ar-H), 8.11 (d, *J =* 8.5 Hz, 2H, Ar-H), 8.32 (s, 1H, CH=N), 12.01 (s, D_2_O exch., 1H, –CONH–); ^13^C-NMR δ ppm: 23.47, 114.00, 117.59, 129.28, 129.46, 132.26 (2C), 135.16 (2C), 136.50, 137.41, 137.52, 148.00, 152.00, 155.52, 168.00; Anal. calcd. for C_20_H_15_BrClN_3_O (427.01): C, 56.03; H, 3.53; N, 9.80. Found C, 56.09; H, 3.76; N, 9.92.

*6-(4-Bromophenyl)-N'-(2,4-dichlorobenzylidene)-2-methylnicotinohydrazide* (**7d**). White crystals (yield 70%), m.p. 269–271 °C; IR (KBr, *ν* cm^−1^): 3413 (NH), 1650 (C=O); ^1^H-NMR δ ppm: 2.67 (s, 3H, CH_3_), 7.55 (d, *J =* 8.50 Hz, 1H, Ar-H), 7.54 (d, *J =* 8.50 Hz, 1H, Ar-H), 7.72 (d, *J =* 8.50 Hz, 2H, Ar-H), 7.96 (d, *J =* 6.50 Hz, 1H, Ar-H), 8.02 (d, *J =* 8.5 Hz, 1H, Ar-H), 8.05 (d, *J =* 8.5 Hz, 1H, Ar-H), 8.11 (d, *J =* 8.5 Hz, 2H, Ar-H), 7.68 and 8.45 (s, 1H, Ar-H), 8.67 (s, 1H, CH=N), 12.01 (s, D_2_O exch., 1H, –CONH); Anal. calcd. For C_20_H_14_BrCl_2_N_3_O (463.15): C, 51.86; H, 3.05; N, 9.07. Found C, 51.95; H, 3.12; N, 9.17.

*6-(4-Bromophenyl)-N'-(4-methoxybenzylidene)-2-methylnicotinohydrazide* (**7e**). White crystals (yield 75%), m.p. 251–253 °C; IR (KBr, ν cm^−1^): 3413 (NH), 1652 (C=O); ^1^H-NMR δ ppm: 2.66 (s, 3H, CH_3_), 3.74 and 3.83 (s, 3H, -OCH_3_), 7.04 (d, *J =* 8.50 Hz, 2H, Ar-H), 7.69 (d, *J =* 8.50 Hz, 2H, Ar-H), 7.72 (d, *J =* 8.50 Hz, 2H, Ar-H), 7.92–7.98 (m, 2H, Ar-H), 8.11 (d, *J =* 8.50 Hz, 2H, Ar-H), 8.26 (s, 1H, CH=N), 11.80 (s, D_2_O exch., 1H, –CONH–); ^13^C-NMR δ ppm: 23.44, 55.79, 114.85, 117.58, 127.00, 129.00, 129.26 (2C), 132.25 (2C), 135.50, 137.00, 137.34, 138.00, 148.26, 156.41, 162.00; Anal. calcd. for C_21_H_18_BrN_3_O_2_ (423.06): C, 59.45; H, 4.28; N, 9.90; Found C, 59.69; H, 4.41; N, 10.12.

*6-(4-Bromophenyl)-2-methyl-N'-(naphthalen-2-ylmethylene)nicotinohydrazide* (**7f**). White crystals (yield 73%), m.p. 273–275 °C; IR (KBr, ν cm^−1^): 3413 (NH), 1648 (C=O); ^1^H-NMR δ ppm: 2.71 (s, 3H, CH_3_), 7.20–8.95 (m, 14H, Ar-H), 8.96 (s, 1H, CH=N), 12.03 (s, D_2_O exch., 1H, –CONH–); Anal. calcd. for C_24_H_18_BrN_3_O (443.06): C, 64.88; H, 4.08; N, 9.46. Found C, 65.07; H, 4.37; N, 9.65.

#### 3.2.4. General Procedure for Preparation of Target Compounds **8a**–**c**

Indoline-2,3-dione derivative **6a**–**c** (1 mmol) was added to a suspension of 6-(4-fluorophenyl)-2-methylnicotinohydrazide (**4c**, 1 mmol) in ethanol (10 mL) and a catalytic amount of glacial acetic acid. The reaction mixture was refluxed for 4 h. The precipitate formed was collected by filtration while hot, washed with hot ethanol, dried and crystallized from ethanol/DMF to furnish compounds **8a**–**c**. The physical properties and spectral data of **8a**–**c** were identical with those reported [40]. The yields of compounds **4a**–**c** ranged from 75%–80%.

### 3.3. Biological Evaluation

#### 3.3.1. Antimycobacterial Activity

The *M. tuberculosis* (RCMB 010126) strain was provided from culture collection of the Regional Center for Mycology and Biotechnology (RCMB), Al-Azhar University (Cairo, Egypt). Isoniazide and pyrazinamide were used as reference drugs. Antimycobacterial activity of the synthesized compounds was evaluated using the microplate Alamar blue assay (MABA) which was performed in black, clear-bottomed, 96 well microplates (in order to minimize background effects). Outer perimeter wells were filled with sterile water to prevent dehydration in experimental wells. Initial compounds dilutions were prepared in dimethyl sulfoxide and subsequent twofold dilutions were performed in the microplates. 0.1 ml of 10^5^ CFU/mL *Mycobacterium tuberculosis* inoculum was added to wells, additional control wells consisted of bacteria only (B). Plates were incubated at 37 °C. Starting at day 4 of incubation, 20 µL of alamarBlue solution (Alamar Biosciences/Accumed, Westlake, OH, USA) and 12.5 µL of 20% Tween 80 were added added to the entire plate. Plates were then incubated at 37 °C, and results were recorded at 24 h post-reagent addition at 590 nm. Percent inhibition was defined as: 1 − (mean of test well/mean of B wells) × 100. Visual MICs were defined as the lowest concentration of drug that prevented a color change.

#### 3.3.2. *In Vitro* Cytotoxic Activity

HT-29 colon cancer and PC-3 prostate cancer cell lines were obtained from the National Cancer Institute (Cairo, Egypt). HT-29 and PC-3, cells were grown in RPMI-1640. Media were supplemented with 10% heat-inactivated FBS, 50 units/mL of penicillin and 50 g/mL of streptomycin and maintained at 37 °C in a humidified atmosphere containing 5% CO_2_. The cells were maintained as a “monolayer culture” by serial subculturing. Cytotoxicity was determined using the SRB method as previously described by Skehan *et al.* [39]. Exponentially growing cells were collected using 0.25% trypsin-EDTA and seeded in 96-well plates at 1000–2000 cells/well in supplemented DMEM medium. After 24 h, cells were incubated for 72 h with various concentrations of the tested compounds as well as doxorubicin as the reference compound. Following 72 h of treatment, the cells were fixed with 10% trichloroacetic acid for 1 h at 4 °C. Wells were stained for 10 min at room temperature with 0.4% SRB dissolved in 1% acetic acid. The plates were air dried for 24 h, and the dye was solubilized with Tris-HCl for 5 min on a shaker at 1600 rpm. The optical density (OD) of each well was measured spectrophotometrically at 564 nm with an ELISA microplate reader (ChroMate-4300, Palm City, FL, USA). The IC_50_ values were calculated according to the equation for Boltzmann sigmoidal concentration-response curve using the nonlinear regression models (Graph Pad, Prism Version 5). The results reported are means of at least three separate experiments. Significant differences were analyzed by one-way ANOVA wherein the differences were considered to be significant at *p* < 0.05.

## 4. Conclusions

In summary, we have synthesized eighteen derivatives based on the nicotinic acid hydrazide scaffold and evaluated their antimycobacterial activity. From the obtained results, it was obvious that condensation of free hydrazides with aldehydes sharply decreased the activity, while isatin hydrazides enhanced the activity. Compounds **8b** and **8c** emerged as the most potent counterparts among all the tested compounds (MIC = 12.5 and 6.25 µg/mL, respectively). They also displayed good drug-likeness scores of 0.62 and 0.41, respectively. The cytotoxicity of hydrazides **8a**–**c** was evaluated against HT-29, PC-3, A549, HepG2 and MCF-7 cancer cell lines. None of the tested hydrazides exhibited cytotoxicity up to 200 µM. The importance of lipophilicity for the antimycobacterial activity was explored via a SAR study. Finally, a theoretical kinetic study was established to predict the ADME of the active derivatives.
